# The value of clinical observation: sleuthing for allergies on the front lines

**DOI:** 10.1186/s13223-022-00716-9

**Published:** 2022-08-24

**Authors:** Elissa M. Abrams, Allan Becker

**Affiliations:** Section of Allergy and Clinical Immunology, Department of Pediatrics and Child Health, University of Manitoba, Winnipeg, Canada

## Commentary

Clinical observation is defined as “the result, answer, judgment or knowledge gained from the act of observing a patient…in healthcare” [1]. Keeping an open mind and learning from patients—the art of clinical observation—has been noted to be “fundamental to the practice of medicine” [2].

We have had an opportunity to review Dr. Gerrard’s fascinating manuscript and are impressed by his observations, or as he states, his anecdotes. Dr Gerrard was one of the first clinicians to practice allergy and epitomizes the utility of clinical observation. He approached patient care with an “N of One” research mind set. As one example, he published the earliest description of the Hygiene Hypothesis in 1976 and was one of the first to suggest a heritable component to atopic disease [3].

Toward the end of his career, he produced a book-length manuscript detailing his many observations on a range of illnesses which he attributed to exposure to foods. The attribution resulted from a variety of approaches from elimination diets to open and even double-blind challenges.

He clearly describes IgE and non-IgE associated disease with some of the best described examples of Food Protein Induced Enterocolitis syndrome (FPIES) pre-dating by decades the recognition of this as a disease entity. He even hints at the role of gut microbiota—an area that is only now beginning to be systematically explored.

While some may dismiss his anecdotal approach to clinical care, we should all be open to the realization that there is much more to be learned in our relatively young specialty. As stated by Shakespeare in Hamlet, “There are more things in heaven and earth, Horatio, Than are dreamt of in your philosophy.” Our understanding of allergic disease has rapidly progressed through the past few decades and continues to do so [4].

From our own experiences it is probable that many of us have seen patients with disease patterns that did not fit a classic explanation. We sometimes can look back and exclaim “I saw that and this (new entity) fits exactly that presentation.” On occasion we do see rare presentations of IgE induced illness. Indeed, our understanding of IgE induced illnesses continues to expand in a variety of areas, most recently in Inflammatory Bowel Disease [5]. More difficult to understand are the immunopathologies of non-IgE or mixed IgE and non-IgE related diseases that sometimes become included in the “Allergy” framework, such as our evolving understanding of eosinophilic esophagitis. These conditions, many of which are described in both this extract and the lengthier manuscript, remain frequently under recognized and misdiagnosed [6]. Evaluation by an allergist when there are concerns about allergic reactions, whether IgE mediated or not, remains the cornerstone of management as the clinical history provides important clues, circling back to the N of one mentality.

We are consultants in Clinical Immunology and Allergy. Our training should provide the very best background from which to explore these complex and oft times frustrating problems (and, yes, patients). While almost all of us are busier than we would ideally choose to be, it is incumbent on each of us to remain curious and open to exploring new concepts. Using an N of One approach to dealing with observations we cannot explain in a satisfactory manner will best serve our patients and our specialty.

To Eat or Not To Eat—That Is the Question

**Sleuthing for Allergies on the Front Lines**

John W. Gerrard, O.C.

D.M. (Oxford), F.R.C.P. (London, England, and Canada) (Deceased)

## Foreword by Chris and Jon Gerrard

Our father, Dr. John Gerrard, was recognized as one of the originators of the “hygiene hypothesis”—that a lack of early childhood exposure to infectious agents increases subsequent susceptibility to allergic diseases by suppressing the natural development of the immune system [3]. More specifically, after studying and comparing IgE antibody levels and the prevalence of allergies in white (Caucasian) and Metis communities in Saskatchewan, he suggested in an August 1976 article in the *Annals of Allergy* that the higher prevalence of allergies in the white community was “the price paid by some members of the white community for their relative freedom from diseases due to viruses, bacteria and helminths.”

The following article, by our father, is shortened from two chapters of a longer, not-yet published book, that he had prepared before he passed away in 2013 about the relationship between foods and a wide range of diseases from asthma, to arthritis, to high blood pressure to a number of brain conditions. While it is clear that many of the reactions to foods which he described in the book are allergies to foods, some like coeliac disease are immune responses and others like withdrawal headaches (such as from caffeine) are food intolerances, but not classic IgE-driven allergy. Rather than get into a debate as to what is an allergic reaction and what is not, it is important to recognize that foods have a remarkable impact on health. This should not be surprising given that foods are among the largest sources of chemical ingredients that a person’s body comes into contact with during a normal day. This alone is a reason for foods and their chemical constituents to play a vital role in health, and for foods to be a trigger of a remarkable proportion of the exposures which trigger allergic and other reactions.

From the early 1950s, our father recognized that foods could be important to health. Together with Horst Bickel and Evelyn Hickmans, he was part of the team that first demonstrated that a low phenylalanine diet was essential for a child with phenylketonuria to be healthy. For lack of a sufficient enzyme to metabolize it, these children experience a buildup of the amino acid phenylalanine which in higher concentrations is neurotoxic, causing extensive brain damage. Our father was the physician on the team who looked after Sheila, the first woman who was given the diet. From this and from his work with children with the then newly described coeliac disease, he became acutely aware of the importance of foods.

In Saskatoon in 1955, there were few patients with PKU or coeliac disease and he focused on more common conditions, in particular, allergies or sensitivities to cow’s milk. Over time he came to appreciate that when he worked with parents to change their children’s diet, he began finding reactions to other foods, including gastro-intestinal reactions.

Our father was a meticulous observer and recorder. He used many approaches to look for links between foods and diseases including single-blind and double-blind experiments. Most important, as he emphasizes in his book, was listening to and learning from the individuals he was helping.

Randomized trials to test the effectiveness of eliminating foods are very difficult when each individual reacts to his/her own individual food intolerances. But double-blind tests on an individual given the food or an alternative carefully disguised to hide the differences between them are possible and he did many. In this way, each person served as their own control.

Finding the offending food in a person with a food allergy or a food intolerance is often difficult and time-consuming, particularly when an individual is skin-test negative to the offending food.

There have recently been major advances in the understanding of non-IgE-mediated food allergy. Notable has been the recent work of Caubet and Abrams [7] describing reactions in children. As Caubet says “Non-IgE mediated gastrointestinal fool allergic disorders (non-IgE-GI-FA) are characterized by subacute or chronic symptoms and classically include food protein-induced enterocolitis syndrome (FPIES), food protein-induced allergic proctocolitis (FPIAP), food protein-induced enteropaty (FPE), celiac disease and cowmilk (CM) induced iron deficiency anemia.” Caubet also notes, “Limited evidence supports the role of food allergens in subsets of constipation, gastro-esophageal reflux disease, irritable bowel syndrome and colic.” As Abrams notes, “Symptoms resolve with elimination of the trigger food from maternal or infant diet.” Caubet comments, “The pathogenesis of non-IgE-GI-FA is poorly understood. These diseases … share eosinophilic dominated inflammation.” (Eosinophils are often associated with allergic conditions.) Abrams notes, “Both conditions (FPIES and FPIAP) are believed to be caused by T-cell mediated inflammation.” In discussing foods and gastrointestinal diseases, our father takes a broad approach to food intolerances and four gastrointestinal diseases. The extent to which food intolerances in these conditions are specifically non-IgE-mediated allergies may vary—but the stories presented are real ones.

### How my interest and concern with food allergies began

I moved from Birmingham, England, to Saskatoon, Saskatchewan, Canada, in July 1955 to become the first Head of the Department of Paediatrics at the newly established clinical medical school at the University of Saskatchewan. Soon after coming to Saskatoon, a colleague and I were invited to speak to a handful of family physicians in Eastend, in the southwest corner of Saskatchewan, on recent advances in the field of medicine. My colleague spoke about the interpretation of electrocardiograms, and was listened to with rapt attention. I spoke about coeliac disease, since I had been involved in recent studies at Birmingham Children’s Hospital which had confirmed that the disease was due to wheat and rye gluten. Although not an allergic condition, coeliac disease is a food intolerance which results from an inflammatory reaction involving T cells which is stimulated in susceptible individuals by exposure to the gliadin protein in gluten. Coeliac disease had been found a number of years earlier to be cured by simply removing wheat and rye from the diet. My audience slept, since they saw so few coeliacs. However, the following day 1 of the family practitioners called on a patient who had recently returned from a well-known clinic in the United States. She was untreated because the clinic did not know the cause of her chronic diarrhoea and wasting disease. She was sent home to die. Unknown to me at the time, her family physician, who had been at my talk, told her that she probably had coeliac disease and would recover if she avoided all wheat and rye products. This she did. Twenty years later, as an incoming nursing student at the University of Saskatchewan, she buzzed me on the intercom while I was doing my rounds, and thanked me for saving her life.

I worked for a while under a well-known paediatrician, Sir Leonard Parsons, who had spent a lifetime studying coeliac disease (CD). At that time there were always several coeliacs on the children’s ward in the hospital. My colleagues and I did not know what caused their illness,

This was the situation when, 1 day in 1950, a Dutch paediatrician, W. K. Dicke, discovered that coeliacs recovered when they were taken off wheat gluten. We immediately took our coeliacs off all wheat products and gave them cream, butter, and bacon; foods we had thought would make them ill. To our surprise they all began to recover. Then we carried out scientific studies to confirm Dicke’s work for ourselves. We now know that it is the gluten, or more correctly gliadin, part of the gluten protein in wheat and rye, and sometimes in oats and barley, that makes these children ill.

We do not know why these changes occur, but it is probably part of an immunological reaction. Coeliacs make antibodies to gliadin, as if purposefully trying to interfere with its absorption.

Englishman Samuel Gee first described coeliac disease in 1888, and 62 years later Dicke discovered the cause of the disease—wheat gluten—and the treatment, a gluten-free diet. What led Dicke to make this discovery was the chance observation of a nurse who noted that in wartime Holland, when rice was not always available, coeliacs seemed to recover when their staple cereal was rice, and relapse when it was wheat.

When I moved from Birmingham to Saskatoon in 1955, I let my new colleagues know that I was interested in seeing patients with coeliac disease. A few were referred to me, but most of the patients did not actually have the disease, even though they had pot-bellies and wasted buttocks. They had all become ill while still on “the bottle,” before they had eaten any cereals, let alone wheat. Most of these children recovered when they were taken off the cow’s milk formulas and given a soya formula, but some didn’t. The sugar in the soya formula was ordinary table sugar, probably derived from sugar cane or beetroot. Many thought that the children improved on the soya formula because it did not contain any lactose, the sugar in breast and cow’s milk. This may have been possible in the exceptional case, but not in the majority, for the children tolerated and thrived on breast milk obtained from our excellent breast milk bank, which contains nearly twice the concentration of lactose present in cow’s milk. Also, some of the cow’s milk-sensitive babies developed diarrhoea when given a soya formula, which contained no lactose. Some were sensitive to other non-lactose containing foods, and when this was the case, the one food that was best tolerated was breast milk. Because these babies did so well on breast milk, it was suspected that their diarrhoea was probably due to a cow’s milk allergy. If this were the case, I expected to find babies with well-recognised allergies, such as eczema and asthma, also due to cow’s milk.

At this time one of my former students asked me to see his 6-month-old baby daughter, Heather, who was wheezing like a chronic asthmatic. I did not want to let my former student down, particularly because his problem was a paediatric one, but I didn’t know how to find out why Heather was wheezing, and so I did what I should have done when I first saw her: I took a careful history.

Heather had been breast-fed until she was 6 months old. Then she was placed on a cow’s milk formula, and soon after she began to wheeze. I took her off the cow’s milk formula and placed her on a soya formula. To my surprise—and relief—she stopped wheezing. Her father, who had never been taught by us in medical school that cow’s milk could cause wheezing, kept putting her back onto the cow’s milk formula, after which she resumed wheezing, until his wife put her foot down and kept Heather on soya.

Two years later, Heather’s mother called to say that she had had another baby daughter, Alice, whom she had breast-fed for 5 months and had then placed on a cow’s milk formula. Like Heather, Alice began to wheeze. She took Alice off cow’s milk and placed her on a soya formula.

I started to be on the lookout for children with other allergies due to cow’s milk. I had no difficulty finding them. But I had a problem: skin tests to cow’s milk were nearly always negative.

This article bristles with observations, or anecdotes if you prefer that term. I know they apply to patients I have seen. To what extent they apply to a wider selection of patients is for others to determine. The realisation that foods, or parts of a food, the gluten component of wheat for example, could actually cause structural changes in the intestinal tract, that cow’s milk could make babies look like coeliacs came slowly. Like a hound following the trail of a fox, I have followed the trail of food allergies and have found it fascinating from a medical point of view. I hope that you will too.

Allergists and patients are lucky that prick tests, which expose mast cells in the skin to a battery of allergens, tell them in many cases what their patients are allergic to. Unfortunately, this led a number of allergists to infer that the patient had no allergies if the skin tests were negative. Whether this is true or not depends on what is meant by the term allergy.

An Austrian doctor, Clemens von Pirquet, coined the term “allergy” long before IgE was discovered, let alone that it was linked to allergies. He defined an allergy as an “altered reaction.” He gave a number of examples, some of which we now know have nothing to do with IgE. Red wine causing a headache, cow’s milk causing nasal congestion, and wheat causing diarrhoea are modern examples that may not be mediated by IgE. It is my experience that the allergens which cause IgE-mediated allergies are relatively easy to identify and relatively straightforward to treat; whereas those that are not mediated by IgE may require more know-how and patience on the part of both doctor and patient.

Fish, peanuts, eggs, and cow’s milk are probably the four most troublesome foods. Some patients are so sensitive to these items that the smell of fish or egg being fried, or of peanuts handled by a fellow passenger on an aeroplane, are sufficient to trigger a severe attack of asthma. These patients know which foods upset them. Prick skin tests to these foods are strongly positive, and they should be avoided. However, many allergies or intolerances to foods are not associated with such positive skin tests and these are much more difficult to identify and to deal with.

### Foods and gastrointestinal diseases

In this section among four significant gastrointestinal disorders I focus on irritable bowel syndrome (IBS)—the others being coeliac disease (CD), Crohn’s disease (CrD), and ulcerative colitis (UC). Evidence that the IBS, CD, and CrD are diet-related is overwhelming, and very suggestive for UC.

However, we should not talk about these diseases without first mentioning Denis Burkitt, an English surgeon who worked in East Africa from 1948 to 1966. He was surprised to find that a number of gastrointestinal diseases which were common in the British in Britain—constipation, appendicitis, stomach ulcers, diverticulitis, and chronic inflammatory bowel diseases such as Crohn’s disease and ulcerative colitis—were rare in the Africans in East Africa. This suggested to him that these diseases might be due to dietary changes associated with modern lifestyles, and in particular to a lack of fibre in Western diets. It is mainly due to Burkitt’s urging that we are now encouraged to eat foods rich in fibre.

### The irritable bowel syndrome (IBS)

IBS is a very common problem that affects the large intestine. It accounts for 10 percent of visits to family physicians, and between 30 and 50 percent of visits to gastroenterologists. Because skin tests typically are negative, and because patients often have other unexplained and unverifiable complaints that cannot be diagnosed through tests, the typical IBS symptoms of abdominal pain, cramping, bloating, gas, and diarrhea and/or constipation are often thought to be due to psychological factors, for which reason patients are often referred to psychiatrists.

Patients with IBS often realize that some foods, milk especially, upset them, and so many are advised to avoid this food. Many are also told to take additional fibre, as Burkitt suggested.

Alun Jones, John Hunter, and their colleagues in Cambridge, England, were among those who prescribed extra fibre. They noticed that though some patients benefited, others did not; in fact, some were made worse. This suggested to them that foods might be responsible for triggering some of the patients’ symptoms.

To find out, twenty-one of Jones and Hunter’s IBS patients participated in a study. For 1 week, they were placed on two simple foods, a meat and a fruit, together with spring or distilled water. Fourteen found that their symptoms cleared. When foods were reintroduced, symptoms returned with the reintroduction of wheat in nine, corn in five, milk and dairy products in four, coffee in four, tea in three, and citrus fruits in two. These results were confirmed by double-blind challenges. It is sad is that even when scientists have gone to these lengths to prove a point, sceptics still don’t believe them.

Alun Jones and John Hunter have continued to treat patients with the IBS along these lines. They have found that virtually any food can trigger symptoms, but the eleven foods most commonly incriminated were wheat (60 percent), corn and milk (44 percent each), cheese (39 percent), coffee (33 percent), rye (30 percent), eggs (26 percent), tea (25 percent), and citrus fruits and barley (24 percent each). The foods best tolerated seem to have been those eaten infrequently, such as raspberries, turkey, fish, and honey.

Finding suitable diets for patients with the IBS is not always easy; most patients are sensitive to several if not many foods. Only 5 percent of the Jones and Hunter study were sensitive to only one food; 28 percent to between two and five foods, 35 percent to between six and ten foods, 17 percent to between eleven and twenty, and 15 percent to more than twenty foods.

Jones and Hunter’s suggestion that foods play a pivotal role in causing the IBS is supported by solid data. Nevertheless, some physicians, like Stephen Bentley for example, feel that psychological factors are important and that the mind rather than the bowel, the psyche rather than the soma, is “irritable.”

Studies carried out by Stephen Bentley were not as rigorous as those by Alun Jones. Diets were not restricted to only two foods, nor was ordinary tap water avoided. Nevertheless, ten of the twenty-one patients who entered his study lost their symptoms on restricted diets. Bentley tried to confirm through double-blind food challenges that their symptoms were actually caused by the foods they *thought* they were sensitive to, but in only three instances was he able to do this to his satisfaction.

Because Bentley thought that psychiatric factors were of overriding importance, psychiatric assessments were made on fourteen of his patients. Twelve had psychiatric problems, nine had neurotic depression, one an anxiety neurosis, a second neurasthenia, and one an affective disorder. Only one patient was assessed psychiatrically both before and after discovering which foods caused her gastrointestinal problems. She was sensitive to yeast. When she avoided yeast, she lost both her gastrointestinal and her psychiatric symptoms, suggesting that both had been triggered by yeast. Four of the five patients with the IBS quoted at the end of this section also had psychiatric or brain related problems, and their irritable bowels and brains responded to dietary measures. Foods can upset both the bowel and the brain, and this is why the IBS has been thought by some to be primarily a psychiatric disorder.

I, like Alun Jones, have found that the IBS is commonly due to foods. It is often easy to sort out which foods because in most instances symptoms clear as soon as the patient avoids all foods. I usually suggest that patients drink only spring water for 1 to 2 days, and if their symptoms clear, to then take single foods for a meal: rice for breakfast, chicken for lunch, carrots for supper, etc. In this way patients are usually able to identify the foods to which they are sensitive. The foods that upset them are then avoided, and those that do not are taken on a rotary basis. If the same food is taken day after day, it too may eventually “irritate” the bowel. Foods that are taken regularly are the foods that tend to trigger a return of symptoms.

If foods were the only cause of the IBS, its treatment would be simple. The gut contains more than semi-digested foods; it also contains bacteria, yeast, and candida. These, too, may trigger symptoms. Interestingly enough, an alteration in bowel flora precipitated by antibiotics may trigger the initial illness, as Alun Jones and his colleagues have pointed out. Such patients are often helped by a combination of dietary management and oral nystatin, an antibiotic used in the treatment of mould infections.

The following case histories illustrate some of the problems encountered by patients with the IBS. The histories are fairly detailed because most patients have a variety of symptoms, and it is only when a full history has been taken that the full extent of their troubles is unravelled.

I will start with the simplest story. Henry was a well-nourished, successful businessman in his early fifties who had a 3-year history of spells of abdominal pain, diarrhoea, bloating, and intermittent fever. Investigations were entirely negative, but he knew something was wrong, but no one knew what it was. He was advised to contact me. In view of the negative findings, I thought that foods might be at the bottom of his troubles. To find out if they were, he would have to avoid all foods, drinking only bottled spring water for 2 to 3 days to see if his symptoms cleared. If they cleared, then he would take one food only at each meal to see which ones upset him.

At the end of 2 days Henry was symptom free. He could hardly believe it. On the third day he felt very hungry, and though I had suggested he should remain on water for 3 days, he decided to eat some chicken. He had no reactions. He then ate a single new food with each meal. He remained perfectly well until he drank milk. Milk and dairy products all caused a return of his troubles. On further questioning he said he had had indigestion for as long as he could remember, but only for the past 3 years had it become intolerable. In retrospect, his history showed that cow’s milk was almost certainly to blame because his symptoms had dated from infancy. Even though milk turned out to be his only problem, it was well that he had had a full gastrointestinal work-up, for he might have had something more serious.

Stephanie was a 19-year-old university student when first seen. She had a much more complicated problem than Henry’s, but it too dated from infancy. As a baby she had been brought up on the breast, and was then symptom free. As soon as foods were introduced her problems started.

Fortunately the relationship between the foods and her reactions were noted, and the foods that upset her were avoided. Eggs caused vomiting, citrus fruits caused hives, melons and bread caused spells of abdominal pain, and bread also caused bloating. Peanuts made her swell up (an anaphylactic reaction), fish caused asthma, and many of the vegetables caused bloating, vomiting, and irritability. She was also sensitive to some inhalants such as cat hair, which made her wheeze.

Many of the foods that upset her gastrointestinal tract also caused what Stephanie described as “cerebral reactions.” Her thoughts would become confused and scrambled, she would be unable to think clearly, and she would become very emotional and unaccountably irritable. As a child these reactions frightened her, and she thought she was losing her mind. In her teens she discovered that it was the foods that were causing both her gastrointestinal and her cerebral reactions. Had she been seen at this time by a psychiatrist, he might well have suggested that all her symptoms were basically psychogenic in origin.

Prick skin tests were strongly positive to cat, peanut, and fish but negative to the other foods that she was sensitive to. The Radioallergosorbent Test (RAST) was strongly positive to peanut and fish but negative to rye, wheat, rice, milk, and potato: all foods that upset her. She had IgE-mediated allergies to peanut and fish, and non-IgE-mediated allergies to the many other foods which she knew upset her. I placed her on an elemental diet. On this, fortunately, all her gastrointestinal and cerebral symptoms cleared. In fact, she felt so well and so alert mentally and happy emotionally that she remained on the elemental formula and ate few other foods for 2 years. She was so suspicious of foods that it was only after much persuasion that she began to test them to find out which did and which did not upset her. She now knows that wild rice, amaranth, quinoa, goat, buffalo, organic pork, and several fruits—apples, pears, plums, cherries and bananas—suit her, and most other foods do not. After a double-blind food challenge with skim milk and placebo, she noted that her cerebral symptoms were triggered by milk but not by the placebo.

As I look back over the patients I have seen with the IBS and the mental symptoms they so often manifest, I am not surprised that the IBS is so frequently thought to be primarily a psychiatric disorder. However, I am intrigued and delighted that both their allergic and psychiatric symptoms have responded so well and so effectively to dietary and environmental control. The easiest patients to treat are those with food-related problems only.

Denis Burkitt was right when he said that the IBS, Crohn’s Disease, and Ulcerative Colitis were due to a Western lifestyle. However, he was mistaken in thinking that the lack of fibre in Western diets was the most important factor. It is foods in general. Why this should be so is a matter for conjecture. The switch from simple, natural foods to the varied modern diet has been more than the gastrointestinal tracts and immune systems of some people can tolerate.

Foods are essential for life. For most people they are one of life’s greatest pleasures. Celebrations demand a feast. I have no desire for my readers to fear the foods they are eating, only to be aware that pain, discomfort, or a dysfunctional body may be due to an allergic sensitivity to certain foods.

Although the diseases and disorders discussed in this article are not always due to foods, the evidence I have presented from my own experience and that of others, has shown that some people with irritable bowels can be made totally well by finding and avoiding the foods that trigger their illnesses. In some instances they also need a safe environment. The advantage of this approach, if it is successful, reduces the load on the health care system and the patient’s out of pocket expenses for medications.

However, this happy state of affairs requires patients to decide to participate in their own recovery, and to count on their own doctor for help and encouragement. The doctor may need more encouragement than the patient.

Every journey starts with a single step; this journey will start with two. The first is Prevention—making sure that children do not fall into the same trap many adults have. The second is Cure—helping those who feel trapped by their allergies. The Western lifestyle has created this trap.

Breast-feeding and breast milk are the best start that can be given to anyone launched into this world of ours. There are many cogent reasons for recommending it.

First of all, breast milk is sterile, served at the right temperature in the right quantities, and supply depends on demand. Its one disadvantage is that it isn’t always available. Mothers may die or be unable to breast feed for a number of reasons. In southern Africa, where my father was a medical missionary from 1915 to 1934, mothers often died in childbirth. There was a taboo against touching babies that survived. My mother offered to look after one such baby. She found a suitable bottle, made a teat from stethoscope tubing, boiled some cow’s milk, diluted it, added a little sugar, and gave it to the abandoned baby whom she called Topsy. The bottle saved Topsy’s life. But as the twentieth century progressed bottle-fed babies became a convenience rather than a necessity. In Europe, upper- and middle-class mothers used to hand over their babies to a wet nurse, but when the bottle became more convenient this old custom died out.

The manufacturers of baby formulas have done their best to emulate Mother Nature. We must thank them for this, but they will never be able to produce a comparable formula, nor will they ever be able to replace the act of breast-feeding and the bonding of infant and mother. Ideally a mother should be free to breast-feed her baby for 6 months. She can start introducing baby food while continuing to offer her breast for a year, or for longer should she wish it.

Experience has taught me to use the dietary approach to help my patients. I suggest that two steps had to be taken to cope with the problems discussed. In the absence of a dietary unit, physicians tend to investigate and diagnose patients without referring them to a dietitian. This works well for coeliac disease because the incriminating foods are well known, but this will not work well for the IBS because the incriminating foods still have to be determined. In these cases the physician should consult a dietitian on elimination diets or fasting to determine which foods should be avoided. When the testing has been completed, an appropriate diet will be prescribed. These studies can nearly always be carried out on outpatients, only occasionally in an ambulatory, self-care facility.

The success of the dietary approach depends as much on the co-operation of the patient as it does on the skill of the dietitian. If patients prefer the traditional approach, no attempt should be made to coerce them. But they should be made aware of an alternative solution. I think that the sooner patients with these disorders are seen, the better. The man with an irritable bowel should not have to wait 40 years to be taken off milk and dairy products.

While the relationship between foods and gastrointestinal disorders is becoming clearer, there remain many areas where my observations suggest it is also important to look.at other organs and organ-systems in the body. One of the last patients I saw around 1995 aptly illustrates this. He was middle aged and well-nourished but had been plagued by troublesome indigestion and unexplained fevers for as long as he could remember. Extensive investigations, carried out locally, had revealed no evidence of any disease. I asked him a few questions, and suspected his troubles were due to foods. I suggested he fast on spring water for 4 days. If his symptoms cleared, he would then have to reintroduce foods one by one to find out which were the troublemakers. “What should I do if this doesn’t help?” he asked. “I’ll have to think again,” I replied. Luckily, he tolerated all foods except cow’s milk and dairy products. He has since been totally well for the first time in his life.

As we move forward in looking at the relationship between foods and other conditions, all I ask of physicians is that they inform their patients that foods can sometimes trigger problems and it might be worthwhile to look into this possibility before launching into a treatment that does not get to the root of the problem.

## Data Availability

No core data available.

## References

[1] Russler D, Liu L, Özsu MT (2009). Encyclopedia of database systems. Clinical observation.

[2] Faustinella F (2020). The power of observation in clinical medicine. Int J Med Educ.

[3] Gerrard JW, Ko CG, Geddes CA, Reggin PL, Gerrard CD, Horne S (1976). Serum IgE levels in white and metis communities in Saskatchewan. Ann Allergy.

[4] Wasserman SI (2000). The allergist in the new millennium. J Allergy Clin Immunol.

[5] Herman SM, Zaborniak K, Bernstein CN (2022). Insight into inflammatory bowel disease pathogenesis: is the answer blowing in the wind?. Inflamm Bowel Dis.

[6] Caubet J-C, Nowak-Węgrzyn A (2011). Current understanding of the immune mechanisms of food protein-induced enterocolitis syndrome. Expert Rev Clin Immunol.

[7] Abrams EM, Hildebrand KJ, Chan ES (2021). Non-IgE-mediated food allergy: evaluation and management. Paediatr Child Health.

